# Surgery in Heart Disease

**Published:** 1902-02-15

**Authors:** 


### SURGERY IN HEART DISEASE.

A PRELIMINARY note, a sort of registration of the idea, is published by Sir Lauder Brunton[1] in regard to the possibility of treating mitral stenosis by surgical means. He points out the absolute hopelessness of extreme conditions of mitral stenosis, questions whether the life led by patients suffering from this condition is not worse even than death, and so leads up to the suggestion that it might be justifiable to propose surgical proceedings for the relief of the obstruction, if by experiments upon animals it could be shown that the necessary operative proceedings are surgically possible. Sir Lauder Brunton has made certain experiments on the dead body in regard to the possibility of reaching and dividing diseased valves, and thus restoring a more free communication between the auricle and the ventricle. He has also, after obtaining a licence for the purpose, tried some experiments on living animals; but, as he remarks, since the carrying out of the treatment, should it ever prove feasible, would have to fall into the hands of surgeons who, before undertaking such a nice bit of work, would certainly have to go through the experimental stage for themselves, he publishes the idea for surgeons to make the most of. In regard to the details of the procedure he suggests that it will be found better to operate from the ventricle than from the auricle, saying that he has often observed in animals—especially during the experiments of the Hyderabad Chloroform Commission—that a needle wound of the ventricle rarely gives rise to any bleeding, whereas even a needle puncture of the auricle may cause much bleeding. Sir Lauder Brunton says that in many experiments which he has made for other purposes he has been astonished at the way in which the heart went on beating, apparently quite unaffected by pulling, compressing, or handling of any kind. He adds that in operating on the living heart the knife should be introduced during diastole, as one is thus less likely to wound the opposite wall of the ventricle. The pericardium should be opened, not merely for convenience of operation, but that it may be left open so as to allow any blood which might ooze out through the ventricular wall to flow away, for the heart has very little power indeed to resist pressure from pericardial effusion. Certainly some very good results have now and again been reported after small wounds of the heart, and it is quite within the bounds of possibility that Sir Lauder Brunton's suggestion may come to something. Unfortunately, one can never feel certain as to the form which the stenosis of this valve has assumed. Where there is a general rigidity and puckering, probably but little good would be done by any operation, but where, as occasionally happens, the valves are attached at their margins and drawn together into a cone, which projects into the ventricle, almost inviting a puncture, there would be a possibility of an absolutely brilliant result if the blood would be kind enough not to clot on the raw surfaces of the incision.

[1] Lancet, Feb. 8.

